# Supplemental Boosting and Cascaded ConvNet Based Transfer Learning Structure for Fast Traffic Sign Detection in Unknown Application Scenes

**DOI:** 10.3390/s18072386

**Published:** 2018-07-22

**Authors:** Chunsheng Liu, Shuang Li, Faliang Chang, Wenhui Dong

**Affiliations:** 1 School of Control Science and Engineering, Shandong University, Ji’nan 250061, China; liuchunsheng@sdu.edu.cn (C.L.); lskzxysdu@gmail.com (S.L.); 2 College of Physics and Electronic Engineering, Dezhou University, Dezhou 253023, China; dongwh_81@163.com

**Keywords:** object detection, traffic sign detection (TSD), transfer learning, adaptive boosting (AdaBoost), convolutional neural networks (CNN)

## Abstract

With rapid calculation speed and relatively high accuracy, the AdaBoost-based detection framework has been successfully applied in some real applications of machine vision-based intelligent systems. The main shortcoming of the AdaBoost-based detection framework is that the off-line trained detector cannot be transfer retrained to adapt to unknown application scenes. In this paper, a new transfer learning structure based on two novel methods of supplemental boosting and cascaded ConvNet is proposed to address this shortcoming. The supplemental boosting method is proposed to supplementally retrain an AdaBoost-based detector for the purpose of transferring a detector to adapt to unknown application scenes. The cascaded ConvNet is designed and attached to the end of the AdaBoost-based detector for improving the detection rate and collecting supplemental training samples. With the added supplemental training samples provided by the cascaded ConvNet, the AdaBoost-based detector can be retrained with the supplemental boosting method. The detector combined with the retrained boosted detector and cascaded ConvNet detector can achieve high accuracy and a short detection time. As a representative object detection problem in intelligent transportation systems, the traffic sign detection problem is chosen to show our method. Through experiments with the public datasets from different countries, we show that the proposed framework can quickly detect objects in unknown application scenes.

## 1. Introduction

The AdaBoost-based detection frameworks have been successfully applied in many real applications of intelligent transportation systems, such as traffic sign detection [1], car detection [2], pedestrian detection [3] and other detection problems [4]. The AdaBoost-based detection methods were originally designed for off-line learning. All training samples have to be available prior to the training process. The trained classifier cannot be dynamically adjusted with new coming samples unless retrained from the beginning, which is time consuming and demands storing all historical samples.

In many vision-based applications, this is a major drawback, as the data may be very different in different application scenes. One off-line trained detector does not have enough generalization ability to adapt to different application scenes. To overcome this problem, Oza [5] proposed an on-line boosting method, which could update strong learners without the need for the storage of samples and retraining the whole classifier. Omar et al. [6] used progressively-improving detectors to update AdaBoost-based detectors online and perform moving object detection. The method in [6] used a small number of training samples to update weights of a detector without selecting new features. Grabner and Bischof [7] improved the method in [5] with a selector-based structure, achieving high performance in object tracking. With a new feature selection process [7], one sample is used to update all weak classifiers and the corresponding voting weight. Instead of a single strong classifier, Chang et al. [8] proposed an online AdaBoost approach for a cascade of strong classifiers. The methods in [6,7,8] can only handle a small training set and are suitable for online tracking and moving object detection problems. Furthermore, these methods lack effective methods for generating a large amount of aligned training samples in on-line testing [6,7,8].

The another shortcoming of the AdaBoost-based detection frameworks is that compared to the convolutional neural network (CNN)-based detection methods, these frameworks have relative lower accuracy and lower generalization ability when handling different application scenes or complex problems. A different boosting method was designed [9] to improve the detection accuracy. The boosted-SVM method [10] was proposed to deal with unbalanced data. Liu et al. [11] utilized a coarse-to-fine tree to improve the detection accuracy of traditional AdaBoost-based detectors. Extreme gradient boosting (XGBoost) [12] is a scalable end-to-end tree boosting system, which can achieve state-of-the-art results on many machine learning challenges.

To overcome these two existing drawbacks, a supplemental training method is proposed to retrain an off-line-trained AdaBoost-based detector; a cascaded convolutional network structure (cascaded ConvNet) is designed and attached at the end of the cascade detector to perform fine detection and verification. The proposed supplemental training method is able to retrain the off-line-trained detector with the false negative samples collected in testing, with the purpose of finding new features to retrain and elongate the off-line-trained detector. The supplemental training method is designed for supplemental training rather than on-line training. Our AdaBoost-based cascade detector, which can be retrained, contains two parts: a basic cascade containing some stages and a supplemental training stage containing some features. The basic cascade is trained to reject most backgrounds and tolerant of nearly all objects. The supplemental training stage is a stage that can be retrained in online testing.

With supplemental training, our retrained AdaBoost-based detector can achieve better generalization ability in different unknown application scenes. Because previous on-line AdaBoost training methods [6,7] did not give a useful method to generate the supplemental training set, we design a method that can generate the supplemental training set. Inspired by the cascaded CNN structure [13], the cascaded ConvNet detector is designed and attached at the end of the AdaBoost-based cascade, with two functions including generating the false negative training set online for supplemental training and accurate detection. The designed cascaded ConvNet detector has a detection net and a calibration net. The detection net is designed to perform fine detection, and the calibration net is designed to align the detected objects to locate and generate aligned training samples accurately for supplemental training.

The main contributions of this study are as follows.

(1) A supplemental training method is proposed to retrain the off-line-trained AdaBoost-based detector. With additional training samples from the application scenes, the retrained AdaBoost-based detector can adapt to different unknown application scenes.
(2) To generate training samples, a cascaded ConvNet detector is designed and attached at the end of the AdaBoost-based cascade. With this cascaded ConvNet detector, the false negatives in testing can be automatically selected and aligned.
(3) For fast and accurate detection in unknown application scenes, we propose a transfer learning-based object detection framework combining the supplemental trained AdaBoost-based detector and the cascaded ConvNet detector. This framework can adapt to different application scenes and achieve a short time consumption.

Similar to the AdaBoost-based methods, the proposed framework is suitable to detect objects with similar structures. This framework is designed to transfer retrain a detector to adapt to unknown application scenes; in this process, a detector from one application scene can be retrained to have the ability to better adapt to other application scenes. This framework is suitable to deal with the same object problem in different applications and is not suitable to transfer a detector for one object to a new detector for another object. For example, it is not suitable to transfer a detector for traffic signs to a new detector for cars.

As a representative object detection problem in intelligent transportation systems, the traffic sign detection problem is chosen to show our method. Through experiments with two public traffic sign datasets, we show that the proposed framework can be retrained to adapt to different unknown application scenes.

The remainder of this paper is organized as follows. Previous traffic sign detection algorithms are reviewed in Section 2. In Section 3, we present our supplemental trained AdaBoost and cascaded ConvNet-based object detection algorithm in detail. In Section 4, our supplemental training and detection algorithms are evaluated by several datasets containing images from different application scenes. The experimental results and their comprehensive analysis and discussion are also included in the same section. Finally, we give the conclusion of this paper and present the future work.

## 2. Related Work

In this study, we chose the traffic sign detection (TSD) problem to show our methods. A brief introduction of the related work is given in this section.

As a key step in traffic sign recognition systems, traffic sign detection (TSD) requires scanning the images or regions of interests (ROIs) to find traffic signs and locate the traffic sign positions. Generally speaking, there are two main methodologies: color-based methods and shape-based methods [14].

Traffic signs usually have standard strong colors to show their shapes and contents. Color segmentation methods are often the first step for the preliminary reduction of the search space, followed by fine detection methods such as geometric edge analysis and shape analysis. The RGB color space is the most intuitive and popular color space. In [15,16], color analysis of the RGB components was performed to detect strong colors such as red, blue and yellow. Transformed from the RGB space, the HSV [17], HIS [18] and Lab [19] color spaces have also been used to extract color regions. These color-based ROI extraction methods often rely on a thresholding process to extract significant color regions and are not robust to sign aging and variations in light reflections. Using more thresholds to perform cascaded color extraction, the cascade-based segmentation [20] is more time-saving and accurate than the thresholding methods on some color spaces. The quadtree-based algorithm [21] uses decision-making tree to extract color spaces. The graph design and visual saliency measure method [22] are used to perform color extraction. As an extraction method combining color enhancement and extreme region extraction, the color maximally stable extremal regions (CMSER) [23] are proposed to find extreme regions in enhanced red and blue color images. The CMSER method is more robust to color changes than the threshold-based extraction methods; yet, this method has inferior performance in extracting other colors and often extracts parts of signs if the color regions are contaminated or asymmetrical.

Traffic signs in different countries usually have formal circular, rectangular, triangular or some other shapes. The shapes and edges are commonly utilized to design TSD methods. Hough transform-based methods are directly used to find circles, rectangles and triangles [24]. Because the Hough transform process is time-consuming, some authors have designed a derivative method of the Hough transform called radial symmetry detection [25,26], which is faster than the Hough transform-based methods. Boumediene et al. [27] designed a detection algorithm for symmetric lines to detect triangular traffic signs. Compared with the shape detection methods, machine learning-based methods are more popular and have better performance. The histogram of oriented gradients (HOG) feature and support vector machine (SVM) classification-based methods [28,29] have been widely used in the TSD problem, because HOG features can express margins and the SVM classifier can classify traffic signs from backgrounds. Hou et al. [30] designed a HOG and SVM classification method for occluded traffic signs, which partly addressed the occluded traffic sign detection problem. The HOG + SVM detection methods have achieved huge success in TSD problem, but these methods often need ROI extraction, which may affect the accuracy of detection. Besides HOG + SVM detection methods, Haar-like features and AdaBoost learning methods [31,32] are utilized to detect traffic signs with special shapes. Boosted cascade tree-based methods [11] are used to detect different types of traffic signs. Unlike HOG + SVM-based detection methods, the boosted cascade methods do not need an ROI extraction process and have been successfully applied in some applications; yet, cascade detectors are often more sensitive to shape changes than the HOG + SVM detector.

In recent years, the CNN-based detection frameworks have achieved high performance in some object detection problems [33,34]. Considering the relatively high computational expense of CNN-based frameworks, the CNN-based methods often rely on GPU platforms to achieve high speed [13]. Though having high detection accuracy, directly using CNN-based frameworks to scan the high-resolution images usually cannot achieve a rapid detection time in real applications.

## 3. Supplemental Trained AdaBoost and Cascaded ConvNet-Based Object Detection

**Figure 1:**
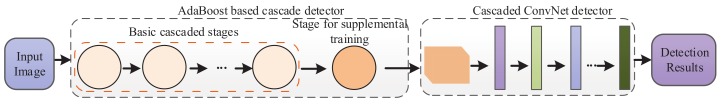
The structure of the proposed detection method.

In this study, a supplemental training method is proposed to perform supplemental training of an off-line trained AdaBoost-based detector. Our AdaBoost-based cascade detector, which can be retrained, contains two parts: a basic cascade containing some stages and a supplemental training stage containing some features. The basic cascade is trained to reject most backgrounds and tolerant of nearly all objects. The supplemental training stage is a stage that can be retrained in online testing. After the basic cascaded stages and the supplemental training stage, a convolutional network-based cascade structure (cascaded ConvNet) is designed and attached at the end of the cascade. The trained cascaded ConvNet detector has two functions including online generation of a false negative training set for supplemental training and performing fine detection. The structure of the proposed detection method is shown in Figure 1. The three main parts are described in the following subsections.

### 3.1. Off-Line-Trained AdaBoost-Based Cascade Detector

**Figure 2:**
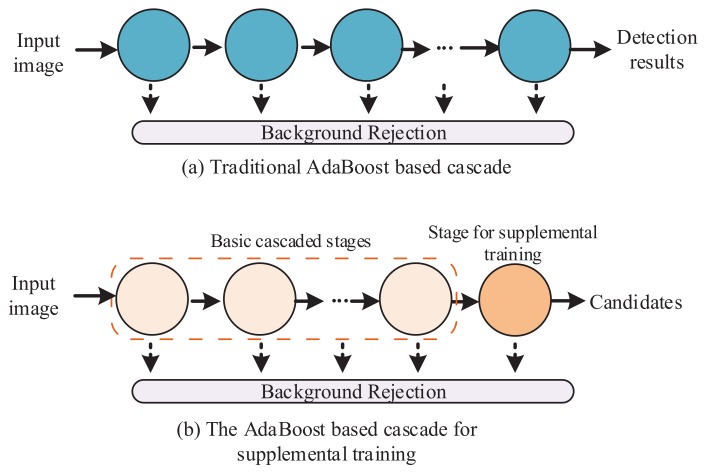
The structure difference between the traditional cascade and our AdaBoost-based cascade for supplemental training.

The off-line-trained AdaBoost-based cascade detector includes some basic cascade stages and a stage of supplemental training. The structure difference between the traditional cascade [35] and our AdaBoost-based cascade for supplemental training is shown in Figure 2. In off-line training, an AdaBoost-based cascade detector with some basic stages and a supplemental training stage is trained. The supplemental training stage can be retrained with our proposed supplemental training method. The training process of the basic cascaded stages is the same as the classical training process in [35]. Following the basic cascaded stages, the supplemental training stage is a trained strong classifier for the purpose of further background rejection. The Haar-like features [35] are used for training. Haar-like features are rectangular features for object detection. They are similar to Haar wavelets and commonly used in some detection problems. The off-line training process is as follows.

Step 1: The basic cascaded stages are trained with the classical training process in [35]. The positive training set is $S_{p}$, and the negative training set is $S_{s}$. The fixed detection rate for each stage is set as 99.9%, and the false alarm rate is set as 50.0%. After this training process, we can obtain a basic cascade with $n_{b}$ stages, with the purpose of rejecting backgrounds and saving detection time. This cascade is trained with enough generalization ability to detect nearly all objects.

Step 2: Following the basic cascade, we use the AdaBoost algorithm to train the supplemental training stage, which is a strong classifier constituted of many weak classifiers. The training set includes the positive training samples, the new added negative samples and the negative samples that cannot be rightly classified with the basic cascade. With the fixed detection rate of 99.9%, we add more weak classifiers to train this strong classifier until the false alarm rate reaches the lowest values. After training, the supplemental training stage with $n_{f}$ features is trained. In training, the principle of adding new negative samples is that the candidate negative samples that cannot be rightly classified by the front stages are added.

### 3.2. Supplemental Training

In online testing, the off-line-trained detector often does not have enough generalization ability to adapt to different application scenes, especially unknown application scenes. Based on experiments, we found that if the false negatives collected in online testing can be used to retrain the AdaBoost-based cascade detector, the detection accuracy should be improved. Based on this observation, we propose a supplemental training method that can retrain the off-line-trained detector for the purpose of achieving stronger generalization ability and higher accuracy.

**Figure 3:**
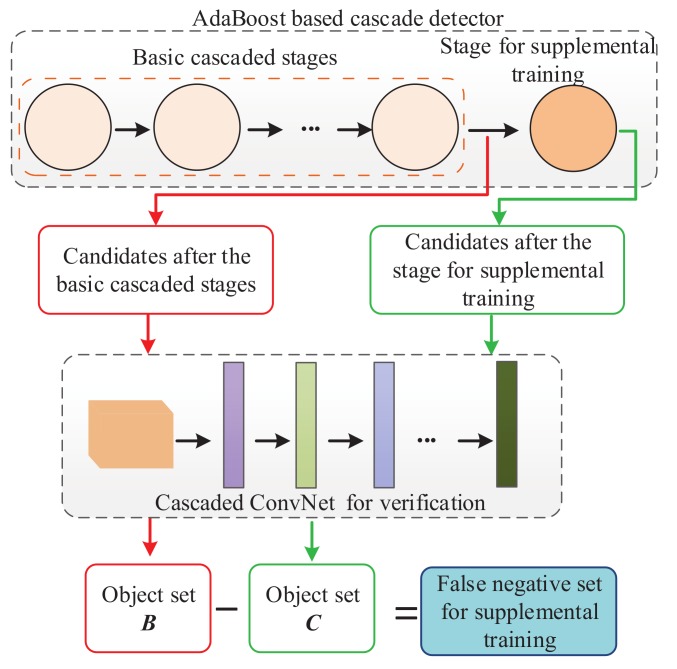
The supplemental training process.

After the AdaBoost-based cascade detector, the false positives can be processed with the following cascaded ConvNet-based detector, whereas the false negatives have been rejected. The cascaded ConvNet-based detector has higher accuracy and better generalization ability than the AdaBoost-based cascade detector. Hence, in this study, we just need to consider the false negatives of the cascade detector. In online testing, we utilize the cascaded ConvNet-based verification method to verify that the samples that have been processed with the AdaBoost-based cascade detector are objects or backgrounds. The flow of the supplemental training process is shown in Figure 3. After verification of the candidates after the basic cascaded stages, the set containing objects is denoted as *B*. After verification of the candidates after the stage of supplemental training, the set containing objects is denoted as *C*. Then, the set of false negatives can be calculated as,

(1) $$S_{a}=B-C.$$

In off-line training of the supplemental training stage, the training set is denoted as $S_{o}$ containing a positive training set $S_{p}$ and a negative training set $S_{s}$. After the off-line training process, the off-line-trained stage is denoted as $H_{o}$, with *T* weak classifiers $C=\{c_{1},c_{2},...,c_{T}\}$, and their weights $D=\{\omega_{1}^{T},\omega_{2}^{T},...,\omega_{n}^{T}\}$. In training of the supplemental training stage, the positive training set is $S_{p}+S_{a}$, and the negative training set is $S_{s}$. There are *n* samples $\left\{x_{i},y_{i}\right\}$ in $H_{o}$ and *m* samples $\left\{x_{i},y_{i}\right\}$ in $S_{a}$. $y_{i}=1$ or 0 is the training label of positives or negatives.

**Figure 4:**
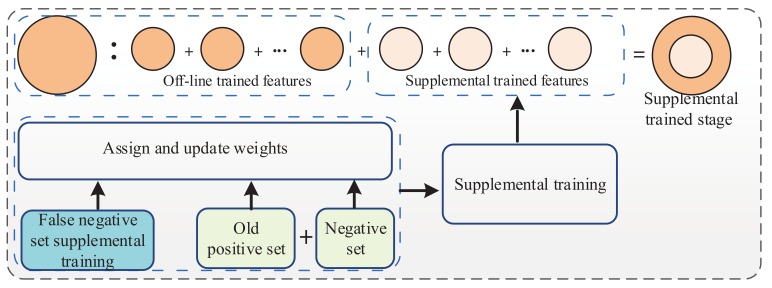
The process of training a supplemental trained stage.

**Figure 5:**
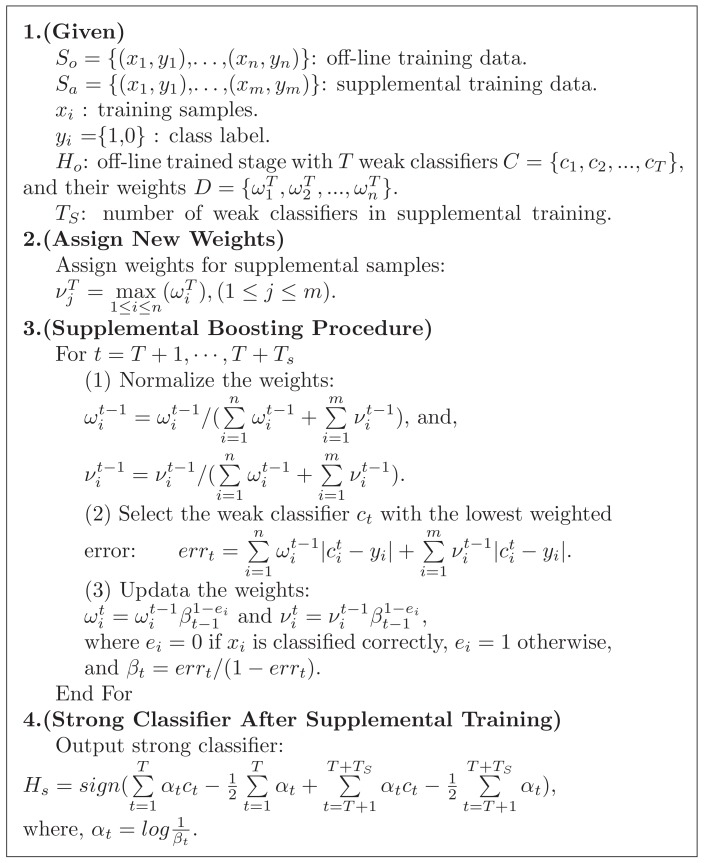
The program pseudo code of the supplemental training method.

The supplemental training has the purpose of finding more features to retrain the off-line-trained detector. The process of training a supplemental trained stage is shown in Figure 4. The false negative set for supplemental training can be obtained from Figure 3. As shown in Figure 4, the false negative set for supplemental training, the old positive set and the negative set are used for supplemental training. The program pseudo code of the supplemental training method is shown in Figure 5. The training process is as follows.

*D* contains the weights after off-line training with *T* iterations. In *D*, the weights of the samples wrongly classified in off-line training have the maximum weights. In supplemental training, the maximum weight is assigned to all samples in $S_{a}$, denoted as,

(2) $$\nu_{j}^{T}=\max_{1\leq i\leq n}\left(\omega_{i}^{T}\right),\left(1\leq j\leq m\right).$$

The supplemental training does the iteration process from the (T+1)-th iteration. Then, all training samples in $S_{a}$ will be mainly considered in the iterations. In the *t*-th iteration, the $\omega_{i}^{t-1}$ and $\nu_{i}^{t-1}$ denote the weights of the samples in $S_{o}$ and $S_{a}$ respectively in the (t-1)-th iteration. The normalized $\omega_{i}^{t-1}$ and $\nu_{i}^{t-1}$ are

(3) $$\omega_{i}^{t-1}=\omega_{i}^{t-1}/\left(\sum_{i=1}^{n}\omega_{i}^{t-1}+\sum_{i=1}^{m}\nu_{i}^{t-1}\right)$$

and

(4) $$\nu_{i}^{t-1}=\nu_{i}^{t-1}/\left(\sum_{i=1}^{n}\omega_{i}^{t-1}+\sum_{i=1}^{m}\nu_{i}^{t-1}\right).$$

The off-line-trained stage $H_{o}$ has *T* weak classifiers. The iteration number of supplemental training is fixed as $T_{s}$. In supplemental training, we need to train to find $T_{s}$ weak classifiers to constitute a strong classifier. The supplemental trained stage $H_{s}$ is expected to have $T+T_{s}$ weak classifiers.

In each boosting process, we select a weak classifier $c^{t}$ with the lowest weighted error,

(5) $$err_{t}=\sum_{i=1}^{n}\omega_{i}^{t-1}c_{i}^{t}-y_{i}+\sum_{i=1}^{m}\nu_{i}^{t-1}c_{i}^{t}-y_{i}.$$

After the weak classifier selection, we need to update the weights of all samples according to the results of the classification

(6) $$\omega_{i}^{t}=\omega_{i}^{t-1}\beta_{t-1}^{1-e_{i}}$$

and

(7) $$\nu_{i}^{t}=\nu_{i}^{t-1}\beta_{t-1}^{1-e_{i}}$$

where $e_{i}=0$ if $x_{i}$ is classified correctly, $e_{i}=1$ otherwise, and $\beta_{t}=err_{t}/\left(1-err_{t}\right)$.

After $T_{s}$ iterations, the strong classifier after supplemental training is,

(8) $$H_{s}=sign\left(\sum_{t=1}^{T}\alpha_{t}c_{t}-\frac{1}{2}\sum_{t=1}^{T}\alpha_{t}+\sum_{t=T+1}^{T+T_{S}}\alpha_{t}c_{t}-\frac{1}{2}\sum_{t=T+1}^{T+T_{S}}\alpha_{t}\right)$$

where $\alpha_{t}=log\frac{1}{\beta_{t}}$.

After the supplemental training, the basic cascaded stages, the retrained supplemental training stage and the cascaded ConvNet are connected together into a new complete detector for object detection. This process needs the training data collected online by the cascaded ConvNet. If the new data that are continually arriving cannot be detected, this system needs to be retrained with these new data. This process can be done with the duplicate architecture of the cascaded ConvNet method.

The samples for supplemental training are often not easy to obtain and have a small number. It is difficult to train a good AdaBoost detector with limited samples. In our study, the old labeled samples are also useful for the new samples, because their structures and appearances do not have much difference. Hence, both the old data and new data are used in the supplemental training process in this study. In supplemental training, if there are enough training samples for supplemental training, the old data can be rejected.

### 3.3. Cascaded ConvNet for Verification and Fine Detection

Some cascaded CNN-based structures have been proposed for fast object detection [13]. Inspired by these methods, we design a cascaded ConvNet-based detector to perform verification and fine detection. The verification process is utilized to generate false negatives for supplemental training. The fine detection process is used for further background rejection and fine detection. The designed cascaded ConvNet detector has a detection net and a calibration net. The detection net is designed to perform fine detection, and the calibration net is designed to align the detected objects for accurate locating and supplemental retraining.

**Figure 6:**
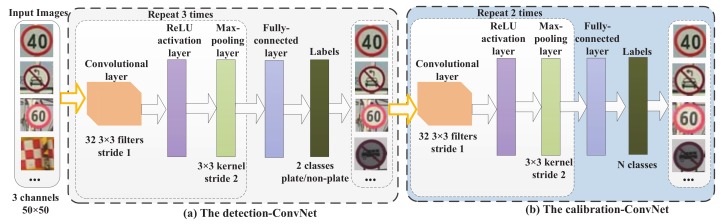
The structure of the cascaded ConvNet-based detector.

After the processing of the AdaBoost-based cascade detector, there is a small number of background subwindows to be rejected. The detection net is trained to detect objects from the remaining candidates. The background samples that cannot be properly classified by the front AdaBoost-based cascade detector are added to the negative training set in training the detection net. As shown in Figure 6, the detection net has three repetitions of the three core layers of a 3×3 convolution layer, a max-pooling layer and a ReLU activation layer and a fully-connected layer and a softmax layer. All input images are resized to 50×50. The R, G and B channels are used in training and testing.

For supplemental retraining, the false negatives need to be aligned. We design the calibration net to adjust the detection windows. The calibration net has two repetitions of the three core layers of a 3×3 convolutional layer, a max-pooling layer and a ReLU activation layer and a fully-connected layer and a softmax layer. The design of this calibration net is similar to the calibration-net design method in [13]. *N* calibration patterns are pre-defined as a set of three-dimensional scale changes and offset vectors ${\left[s_{n},x_{n},y_{n}\right]}_{n=1}^{N}$. Given a detection window (x,y,w,h) with the top-left corner at (x,y) of size (w,h), the calibration pattern adjusts the window to be

(9) $$(x-\frac{x_{n}w}{s_{n}},y-\frac{y_{n}w}{s_{n}},\frac{w}{s_{n}},\frac{h}{s_{n}}).$$

After the detection of the previous cascade detector, the detection windows may have deviations of the coordinate *x* and the width *w*; Formula (9) is designed to change *x* and *w* for calibration. In this work, the calibration net has N=27 patterns formed by all combinations of,

(10) $$\begin{array}{c} s_{n}\in \left\{1,1.10,1.21\right\},\\ x_{n}\in \left\{-0.17,0,0.17\right\},\\ y_{n}\in \left\{-0.17,0,0.17\right\}. \end{array}$$

Given a detection window, the region is resized to 50×50 as the input image for the calibration net. The calibration net outputs a vector of confidence scores $\left[c_{1},c_{2},...,c_{N}\right]$. The average results of the patterns of high confidence score are chosen as the adjustment [s,x,y], i.e.,

(11) $$\left[s,x,y\right]=\frac{1}{Z}\sum_{n=1}^{N}\left[s_{n},x_{n},y_{n}\right]I\left(c_{n}>t\right)$$

where

(12) $$Z=\sum_{n=1}^{N}I\left(c_{n}>t\right)$$

and

(13) $$I\left(c_{n}>t\right)=\left\{\begin{array}{cc} 1, & c_{n}>t\\ 0, & \mathrm{otherwise}. \end{array}\right.$$

Here, *t* is a threshold to filter out low confidence patterns.

After processing with the detection net and the calibration net, the detection windows of object are extracted. The cascaded ConvNet can be used for fine detection, achieving high accuracy in both detection and localization. The cascaded ConvNet can also be used for verification to get supplemental training samples. In these experiments, we create a set of options for training networks using stochastic gradient descent with momentum. We set the initial learning rate as 0.001 and reduced the learning rate by a factor of 0.1 every eight epochs; we set the maximum number of epochs for training to 40 and use a mini-batch with 128 observations at each iteration.

## 4. Experiments and Results

### 4.1. Dataset and Setup

**Figure 7:**
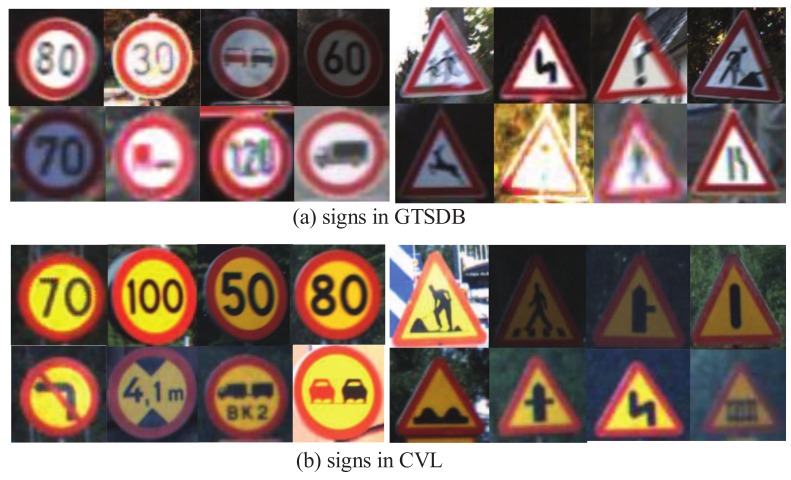
The signs from GTSRB and CVL.

The proposed methods are evaluated on two public traffic sign datasets from different countries including the German GTSRB (German Traffic Sign Recognition Benchmark) dataset [36] and the Swedish CVL (Computer Vision Laboratory) dataset [37]. In intelligent transportation systems, it is common to transfer a detector from one application scene to other unknown application scenes. The GTSRB dataset has more than 50,000 signs in total, which is a large comprehensive dataset covering different types of German traffic signs. The CVL dataset has more than 20,000 images, which is a dataset for testing the Swedish traffic sign detector. A shown in Figure 7, the traffic signs in Germany and Sweden have similar structures, but different colors. These two datasets are suitable to evaluate the proposed transfer learning method on different unknown application scenes.

We used 8320 circular signs and 8000 triangular signs in the GTSRB dataset to train a circular detector and a triangular detector, respectively. The 9793 images with 3750 circular signs and 439 triangular signs in CVL (CVL-A ) are used for testing the off-line-trained detectors before supplemental training. With supplemental training sets, we can retrain the detectors with our supplemental training method. In the supplemental training, two thirds of the samples are used for training, and the rest are for validation. In testing, the 9363 images in the CVL dataset (CVL-B) with 2297 circular signs and 388 triangular signs are tested as images from unknown application scenes and are utilized to test our supplemental trained detectors. The detailed description of these datasets is shown in Table 1.

In these experiments, we do not know the application scenes initially in the off-line training process. These experiments can evaluate the adaptive capacity of our method on unknown application scenes by supplemental training. In testing, to achieve scale invariance, the input image is continuously scaled with a scale *c* into a series of images. In this study, the detector with scaling parameter $c=\frac{1}{1.10}$ can detect traffic signs at different resolutions. Scan the scaled images at step *s* with the 30×30 detector. *s* is 5 pixels in these experiments. The detector can scan the image at different scales and different positions. The codes are programmed with VC++, OpenCV, MATLAB on a PC with an Intel i7-7700 CPU, 8 GB RAM and an NVIDIA GTX 1060 GPU with 6 GB RAM. The AdaBoost-based cascade detector is programmed with VC++ and OpenCV. The cascaded ConvNet-based verification and fine detection method are programmed with MATLAB.

### 4.2. Evaluation of the Supplemental Boosting Method

The experiment in this part was designed to demonstrate that the proposed supplemental training method can retrain the off-line-trained AdaBoost-based detector, achieving better generalization ability. In this experiment, we tested the performance of the proposed supplemental training method by transferring a detector for GTSRB to a new detector for CVL.

**Figure 8:**
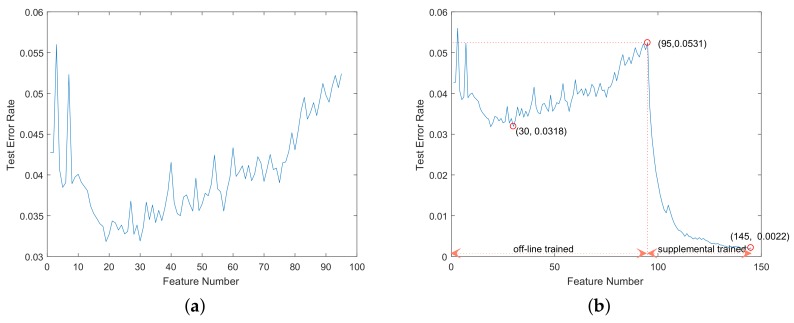
The curves of the feature number and the test error rate on circular sign detection. The curve in (**a**) is the performance of the off-line-trained AdaBoost-based detector. The curve in (**b**) is the performance of the supplemental trained AdaBoost-based detector. The three red circle markers in the curves denote the test error rates of the basic cascaded stages, the stage of supplemental training and the supplemental trained detector, respectively.

**Figure 9:**
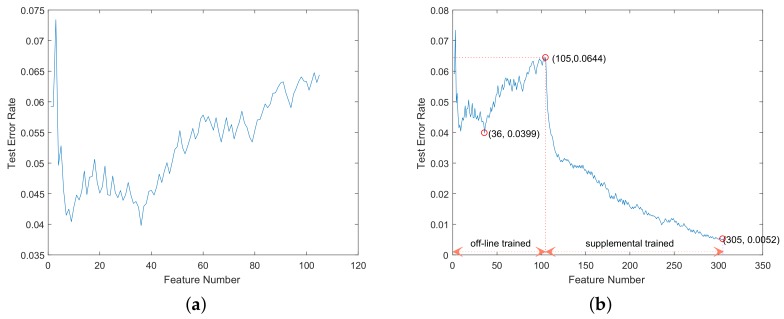
The curves of the feature number and the test error rate on triangular sign detection. The curve in (**a**) is the performance of the off-line-trained AdaBoost-based detector. The curve in (**b**) is the performance of the supplemental trained AdaBoost-based detector.

In our traffic sign detector, the basic cascaded stages contained 30 features and 36 features for circular and triangular signs, respectively, the training goal of which was to achieve the highest detection rate in off-line training. The off-line-trained stage of supplemental training contained 65 features and 69 features, respectively, the training goal of which was to achieve a detection rate greater than 99.5% and an as low as possible false alarm rate. Before supplemental training, both the basic cascaded stages and the stage of supplemental training were off-line trained. We tested the off-line trained detectors on CVL-B. The curves of the feature number and the test error rate are shown in Figure 8a and Figure 9a. The test error rate in this experiment was defined as the ratio of the false negative number and the positive number.

In this test, the triangular signs were mirrored or affine transformed to get four-times the number of samples. Using the training samples from CVL-A, we retrained the off-line-trained detectors with the proposed supplemental boosting method. The supplemental trained stage contained 50 features and 200 features, respectively, the goal of which was to achieve the lowest test error rates. The supplemental trained detectors were tested on CVL-B. The curves of the feature number and the test error rate are shown in Figure 8b and Figure 9b. In Figure 8b and Figure 9b, the off-line trained part of the curves are the performance of the off-line-trained detectors including basic cascaded stages and the stage of supplemental training. The three red circle markers in the curves denote the test error rates of the basic cascaded stages, the stage of supplemental training and the supplemental trained detector, respectively.

As shown in Figure 8 and Figure 9, the off-line-trained detectors had bad generalization ability in detecting signs from unknown application scenes, achieving 0.0531 and 0.0644 error rates on detecting circular and triangular signs, respectively (marked by the second circle markers in Figure 8b and Figure 9b). The supplemental trained detectors can rapidly reduce the error rates with supplemental training features, achieving 0.0022 and 0.0052 error rates on testing circular and triangular signs, respectively (marked by the third circle markers in Figure 8b and Figure 9b). Without any training samples form the application scenes, the off-line-trained detectors cannot achieve low error rates. The proposed supplemental training method utilized the false negatives from the application scenes to perform supplemental training of the detector and can achieve much lower error rates. Hence, these experimental results showed that the proposed supplemental training method can improve the accuracy and the generalization ability of the off-line-trained detectors.

### 4.3. Evaluation of the Cascaded ConvNet Method

As the last detection process in our hybrid cascade, the cascaded ConvNet detector had two functions, including performing fine detection of the remaining candidates and generating aligned training samples for supplemental AdaBoost training. The experiment in this part was designed to evaluate that without any training samples from the application scenes, the proposed cascaded ConvNet detector can achieve good performance in both detection and localization. The test samples were from the detected samples before and after the stage of supplemental training.

Unlike the AdaBoost-based learning method, the ConvNet-based learning method can deal with a large amount of training samples and can deal with samples that are not well aligned. For the cascaded ConvNet training, we used mirroring, small position translation, pixel value shift and size scaling to get more than ten-times the training samples.

To demonstrate that the designed cascaded ConvNet detector had good performance in both detection and localization, we compared the cascaded ConvNet detector to three other machine learning-based detection methods including the HOG + SVM detector [28], the Haar-like + AdaBoost detector [31] and the CNN detector [33]. The statistical results before and after the stage of supplemental training are shown in Table 2 and Table 3, respectively.

The parameters of the detection rate (DR), false alarms per image (FAPI) and intersection-over-union (IOU) were used to evaluate the detection results. The DR is the ratio of the true positive number to the number of all positives after the basic cascaded stages. The FAPI is the number of false alarms per image. The IOU is defined as,

(14) $$IOU=\frac{A⋂B}{A⋃B}$$

where *A* and *B* are the regions of the detection result and the ground truth, respectively.

The statistical results in Table 2 show that dealing with the detected samples before the stage of supplemental training, the proposed cascaded ConvNet achieved the highest DRs of 98.8% and 98.7%, the lowest FAPI of 0.16 and the highest IOU of 87.2%. With trials of different HOG features, the chosen HOG features can be combined with a linear SVM classifier to achieve relatively high DRs of 93.6% and 92.3% and the largest FAPI value of 2.00. Without training samples from CVL, the Haar-like + AdaBoost detector had bad performance in classifying license plates from these backgrounds, achieving 92.3% and 88.4% DRs. Achieving similar DRs with CNN, our method had a 8.3% higher IOU and a 0.08 lower FAPI than those of the faster regions with CNN (RCNN) detector. Similar to the analysis of the results in Table 2, the statistical results in Table 3 show that dealing with the detected samples after the stage of supplemental training, the proposed cascaded ConvNet achieved 100.0% DRs in circular and triangular sign detection and the lowest FAPI of 0.09 and the highest IOU of 91.0%, which were much better than the other methods. From the analyses of the results in Table 2 and Table 3, it can be concluded that the proposed cascaded ConvNets can achieve high DR, low FAPI and high IOU values before or after the stage of supplemental training, which ensures that the proposed cascaded ConvNets method can realize the two functions of performing fine detection and generating supplemental training samples.

There are three main reasons for this achievement. Firstly, with a calibration-ConvNet, the proposed cascaded ConvNet can align the detection results to achieve high IOU values. Secondly, the cascade structure can efficiently reject background subwindows in different cascaded parts, achieving low FAPI values. Thirdly, the license plates aligned with the calibration-ConvNet can be more easily classified with the back-ConvNet, achieving better performance in both DR and FAPI. Based on the analyses, it can be concluded that without any training samples from the application scenes, the proposed cascaded ConvNet can achieve high performance in both detection and localization. Hence, the proposed cascaded ConvNet can effectively perform fine detection of the remaining candidates or generate aligned training samples for supplemental AdaBoost training.

### 4.4. Performance Evaluation of the Proposed Detector

In this part, we design experiments to demonstrate the hypothesis that the proposed supplemental trained AdaBoost and cascaded ConvNet-based object detection algorithm has the ability to quickly and accurately detect traffic signs in unknown application scenes. The methods for comparison included the Haar-like + AdaBoost detection method [31], the SFC-tree (Split Flow Cascade tree) + AdaBoost detection method [11], the CMSER + SVM detection method [23] and the faster RCNN (faster regions with CNN) detection method [33].

All these detectors in this comparison were off-line trained without using any training samples from the test scenes. The proposed method can be retrained with the new data. The Haar-like + AdaBoost detector is the classical cascade detector based on Haar-like features and the AdaBoost method. The SFC-tree + AdaBoost detection method is an AdaBoost-based coarse-to-fine tree detector. The CMSER + SVM detector uses CMSER (color maximally stable extremal regions) to extract regions of interest and then uses the SVM classifier to perform the detection. Being proposed to detect different types of objects, the faster RCNN detector was trained to detect traffic signs in this comparison.

For our detector, the off-line-trained AdaBoost-based detector of circular signs had seven basic stages and a supplemental stage containing 95 features in total; the off-line-trained detector of triangular signs had seven basic stages and a supplemental stage containing 105 features in total. The supplemental trained detectors of circular signs and triangular signs had 50 features and 200 features, respectively. The trained cascaded ConvNet detector had a detection net and a calibration net as described in the Section 3.3.

The statistical results of the comparison are shown in Table 4. The detection results were reflected in three parameters: precision, recall, and detection time. The detection time is the average consumed time in sign detection per image. The parameters of precision and recall are defined as

(15) $$Precision=\frac{TP}{TP+FP}$$

and

(16) $$Recall=\frac{TP}{TP+FN}$$

where TP is the number of true positives, FP the number of false positives and FN the number of false negatives.

From Table 4, it can be seen that the proposed supplemental trained AdaBoost + CNN detector can achieve the highest precision value of 97.52% and the second highest recall value of 97.02% on detecting circular and triangular signs. Without using any training samples from the test scenes, the off-line-trained AdaBoost-based detectors [11,31] achieved low precision values and low recall values. With a color-based ROI extraction process, the CMSER + SVM-based detector [23] achieved relatively high precision and recall values for detecting signs. For detecting all signs, the precision of our method was 0.65% higher than that of the faster RCNN, and the recall of our method was 1.04% lower than that of the faster RCNN. A high precision value means a small false positive number. The 0.65% higher precision value means that the false positive number of our method was 19 less than that of the faster RCNN. The 1.04% lower recall means that the number of our undetected objects was 28 less than that of the faster RCNN.

**Figure 10:**
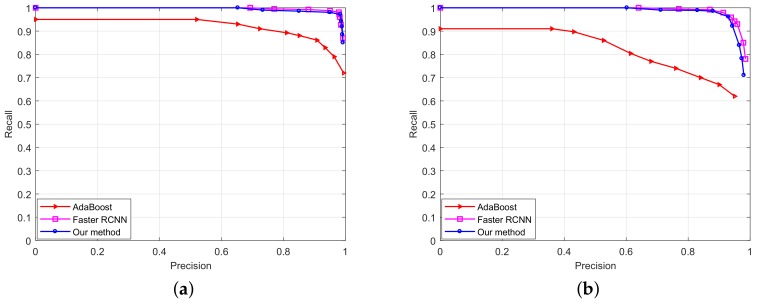
The ROC curves of AdaBoost, faster faster regions with CNN (CNN) and the proposed method: (**a**) shows the ROC curves of these three methods tested on circular signs; (**b**) shows the ROC curves of these three methods tested on triangular signs.

The methods of AdaBoost, faster RCNN and the proposed method in this comparison can obtain the ROC curves. The ROC curves of these three methods are shown in Figure 10. The ROC curves in (a) and (b) show that the faster RCNN and the proposed method had similar performance tested on circular signs or triangular signs, which was much better than that of AdaBoost. Shown in Table 4, achieving similar precision and recall values, the faster RCNN detector needed a 5.2-s detection time, which is too slow to apply in real applications, whereas our method needed a 260-ms detection time, which is approximately one twentieth of that of the faster RCNN. Hence, achieving similar recalls and precisions, the proposed detector can achieve twenty-times faster processing speed than that of the faster RCNN.

**Figure 11:**
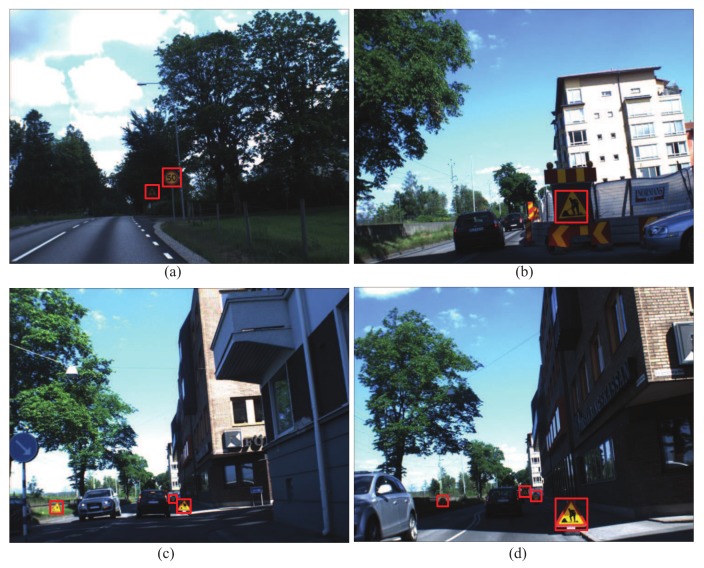
Part of our detection results. The images from (**a**–**d**) are the detected results from CVL dataset.

The achievements of our method were mainly due to the following reasons. Firstly, the supplemental training method can retrain the off-line-trained AdaBoost detector to achieve high precisions and recalls in different unknown application scenes. Secondly, the AdaBoost-based detector can rapidly perform coarse detection and save the detection time of the following cascaded ConvNet detector. Lastly, we trained and attached a cascaded ConvNet-based detector to perform fine detection and further background rejection achieving high precisions and recalls. With these improvements, the proposed detector can achieve the highest precision of 97.52%, the second highest recall of 97.02% and the small consumption time of 286 ms. The experimental results show that the proposed detector can achieve high accuracy, fast detection time and strong generalization ability in detecting objects in different unknown application scenes. Part of our detection results is shown in Figure 11.

## 5. Conclusions

Several novel methods have been proposed to construct a supplemental trained AdaBoost and cascaded ConvNet-based object detection structure. The presented detection structure can rapidly detect objects in unknown application scenes, achieving high precision and recall.

To address the problem of supplemental training of an AdaBoost-based detector, we propose a supplemental training method that is able to retrain the off-line-trained detector with the false negative samples collected in testing. To improve the detection accuracy and online collection of supplemental training samples, a cascaded ConvNet is designed and attached at the end of the AdaBoost-based detector. The cascaded ConvNet detector is designed with a detection net and a calibration net. The detection net is designed to perform fine detection, and the calibration net is designed to align the detected objects for accurately locating and generating supplemental training samples.

The traffic sign detection problem is chosen to show our method. Through experiments with two datasets from different countries, we show that the proposed framework can be retrained to adapt to unknown application scenes in real applications. This structure can be easily extended to other object detection problems. As future research work, we aim to improve this structure in order to apply it to other object detection problems.

## Figures and Tables

**Table 1:** Details of the training and testing datasets from GTSRB and CVL.

Dataset	Details of the Dataset						
	Purpose	Resolution	Number of Images	Sign Height	Number of Circular Signs	Number of Triangular Signs	Number of All Signs
GTSRB	Training off-line detector	30 × 30	16,320	30 pixels	8320	8000	16,320
CVL-A	Testing before supplemental training	1280 × 960	9793	30∼180 pixels	3750	439	4189
CVL-B	Testing after supplemental training	1280 × 960	9363	30∼180 pixels	2297	388	2685

**Table 2:** Performance of different detection methods before the stage of supplemental training. DR, detection rate; FAPI, false alarms per image; IOU, intersection-over-union.

Methods	DR		FAPI	IOU
	Circular	Triangular		
HOG + SVM [28]	93.6%	92.3%	2.00	76.3%
Haar-like + AdaBoost [31]	92.3%	88.4%	1.65	78.4%
CNN [33]	98.7%	98.7%	0.24	78.9%
Cascaded ConvNets	98.8%	98.7%	0.16	87.2%

**Table 3:** Performance of different detection methods after the stage of supplemental training.

Methods	DR		FAPI	IOU
	Circular	Triangular		
HOG + SVM [28]	97.4%	96.5%	0.90	78.5%
Haar-like + AdaBoost [31]	93.1%	82.0%	0.65	81.3%
CNN [33]	100.0%	100.0%	0.18	80.7%
Cascaded ConvNets	100.0%	100.0%	0.09	91.0%

**Table 4:** Performance of different detection methods.

Detection Methods	Performance						
	Recall (Cir)	Precision (Cir)	Recall (Tri)	Precision (Tri)	Recall (All)	Precision (All)	Time
Haar-like + AdaBoost [31]	86.11% (1978/2297)	90.78% (1978/2179)	80.41% (312/388)	61.42% (312/508)	85.29% (2290/2685)	85.23% (2290/2687)	141 ms (CPU)
SFC-tree + AdaBoost [11]	86.33% (1983/2297)	90.63% (1983/2188)	80.41% (312/388)	63.29% (312/493)	85.47% (2295/2685)	85.60% (2295/2681)	107 ms (CPU)
CMSER + SVM [23]	88.77% (2039/2297)	92.64% (2039/2201)	85.57% (332/388)	70.34% (332/472)	88.31% (2371/2685)	88.70% (2371/2673)	93 ms (CPU)
Faster RCNN [33]	98.09% (2253/2297)	97.87% (2253/2302)	97.94% (380/388)	91.34% (380/416)	98.06% (2633/2685)	96.87% (2633/2718)	5.2 s (CPU + GPU)
Our method	97.17% (2232/2297)	98.32% (2232/2270)	96.13% (373/388)	93.02% (373/401)	97.02% (2605/2685)	97.52% (2605/2671)	260 ms (CPU + GPU)

## Abbreviations

TSD	Traffic sign detection
AdaBoost	Adaptive boosting
CNN	Convolutional neural networks
Cascaded ConvNet	Cascaded convolutional network structure
CMSER	Color maximally stable extremal regions
SVM	Support vector machine
HOG	Histogram of oriented gradients
RGB	Red, green, blue
HSV	Hue, saturation, value
HIS	Hue, saturation, intensity
ROI	Region of interest

## References

[1] Baró X., Escalera S., Vitri J., Pujol O., Radeva P. (2009). Traffic sign recognition using evolutionary AdaBoost detection and forest-ECOC classification. IEEE Trans. Intell. Transp. Syst..

[2] Xu Y., Yu G., Wang Y., Wu X., Ma Y. (2016). A Hybrid Vehicle Detection Method Based on Viola-Jones and HOG + SVM from UAV Images. Sensors.

[3] Guo L., Ge P., Zhang M., Li L., Zhao Y. (2012). Pedestrian detection for intelligent transportation systems combining AdaBoost algorithm and support vector machine. Expert Syst. Appl..

[4] Luo L., Tang Y., Zou X., Wang C., Zhang P., Feng W. (2016). Robust Grape Cluster Detection in a Vineyard by Combining the AdaBoost Framework and Multiple Color Components. Sensors.

[5] Oza N., Russell S. Online bagging and boosting. Proceedings of the 2005 IEEE International Conference on Systems, Man and Cybernetics.

[6] Javed O., Ali S., Shah M. Online Detection and Classification of Moving Objects Using Progressively Improving Detectors. Proceedings of the CVPR.

[7] Grabner H., Bischof H. On-line Boosting and Vision. Proceedings of the 2006 IEEE Computer Society Conference on Computer Vision and Pattern Recognition.

[8] Chang W., Cho C. (2010). Online Boosting for Vehicle Detection. IEEE Trans. Syst. Man Cybern. Syst..

[9] Huang C., Ai H., Li Y., Lao S. (2007). High-performance rotation invariant multiview face detection. IEEE Trans. Pattern Anal. Mach. Intell..

[10] Ziȩba M., Tomczak J.M. (2015). Boosted SVM with active learning strategy for imbalanced data. Soft Comput..

[11] Liu C., Chang F., Chen Z. (2014). Rapid multiclass traffic sign detection in high-resolution images. IEEE Trans. Intell. Transp. Syst..

[12] Chen T., Guestrin C. XGBoost: A Scalable Tree Boosting System. Proceedings of the 22nd SIGKDD Conference on Knowledge Discovery and Data Mining.

[13] Li H., Lin Z., Shen X., Brandt J., Hua G. A Convolutional Neural Network Cascade for Face Detection. Proceedings of the 2015 CVPR.

[14] Møgelmose A., Trivedi M.M., Moeslund T.B. (2012). Vision-based traffic sign detection and analysis for intelligent driver assistance systems: Perspectives and survey. IEEE Trans. Intell. Transp. Syst..

[15] Broggi A., Cerri P., Medici P., Porta P., Ghisio G. Real time road signs recognition. Proceedings of the 2007 IEEE Intelligent Vehicles Symposium.

[16] Andrey V., Kang-Hyun J. Road guidance sign recognition in urban areas by structure. Proceedings of the 2006 International Forum on Strategic Technology.

[17] Marinas J., Salgado L., Arróspide J., Nieto M. Detection and tracking of traffic signs using a recursive Bayesian decision framework. Proceedings of the 2011 14th International IEEE Conference on Intelligent Transportation Systems (ITSC).

[18] Jau U.L., Teh C.S., Ng G.W. A comparison of RGB and HSI color segmentation in real-time video images: A preliminary study on road sign detection. Proceedings of the 2008 International Symposium on Information Technology.

[19] Khan J.F., Bhuiyan S.M.A., Adhami R.R. (2009). Image segmentation and shape analysis for road-sign detection. IEEE Trans. Intell. Transp. Syst..

[20] Deguchi D., Shirasuna M., Doman K. Intelligent traffic sign detector: Adaptive learning based on online gathering of training samples. Proceedings of the IEEE Conference Intelligent Vehicles Symposium.

[21] Ruta A., Porikli F., Watanabe S., Li Y. (2011). In-vehicle camera traffic sign detection and recognition. Mach. Vis. Appl..

[22] Yuan X., Guo J., Hao X., Chen H. (2015). Traffic Sign Detection via Graph-Based Ranking and Segmentation Algorithms. IEEE Trans. Syst. Man Cybern. Syst..

[23] Greenhalgh J., Mirmehdi M. (2012). Real-time detection and recognition of road traffic signs. IEEE Trans. Intell. Transp. Syst..

[24] Morse B.S. (2000). Segmentation (Edge Based, Hough Transform).

[25] Barnes N., Zelinsky A., Fletcher L.S. (2008). Real-time speed sign detection using the radial symmetry detector. IEEE Trans. Intell. Transp. Syst..

[26] Loy G., Barnes N. Fast shape-based road sign detection for a driver assistance system. Proceedings of the 2004 IEEE/RSJ International Conference on Intelligent Robots and Systems (IROS).

[27] Boumediene M., Cudel C., Basset M., Ouamri A. (2013). Triangular traffic signs detection based on RSLD algorithm. Mach. Vis. Appl..

[28] Wang G., Ren G., Wu Z., Zhao Y., Jiang L. A robust coarse-to-fine traffic sign detection method. Proceedings of the the 2013 International Joint Conference on Neural Networks (IJCNN).

[29] Salti S., Petrelli A., Tombari F. A traffic sign detection pipeline based on interest region extraction. Proceedings of the the 2013 International Joint Conference on Neural Networks (IJCNN).

[30] Hou Y., Hao X., Chen H. (2017). A Cognitively Motivated Method for Classification of Occluded Traffic Signs. IEEE Trans. Syst. Man Cybern. Syst..

[31] Segvic S., Brkic K., Kalafatic Z. (2014). Exploiting temporal and spatial constraints in traffic sign detection from a moving vehicle. Mach. Vis. Appl..

[32] Timofte R., Zimmermann K., Gool L.V. (2014). Multi-view traffic sign detection, recognition, and 3D localisation. Mach. Vis. Appl..

[33] Ren S., He K., Girshick R., Sun J. (2017). Faster R-CNN: Towards Real-Time Object Detection with Region Proposal Networks. IEEE Trans. Pattern Anal. Mach. Intell..

[34] Zhong J., Lei T., Yao G. (2017). Robust Vehicle Detection in Aerial Images Based on Cascaded Convolutional Neural Networks. Sensors.

[35] Viola P., Jones M.J. (2004). Robust real-time face detection. Int. J. Comput. Vis..

[36] Houben S., Stallkamp J., Salmen J., Schlipsing M. Detection of traffic signs in real-world Images: The German Traffic Sign Detection Benchmark. Proceedings of the the 2013 International Joint Conference on Neural Networks (IJCNN).

[37] Larsson F., Felsberg M. (2011). Using Fourier Descriptors and Spatial Models for Traffic Sign Recognition. Lect. Notes Comput. Sci..

